# Microfluidic Production of Exosome-Mimicking Lipid Nanoparticles for Enhanced RNA Delivery: Role of Exosomal Proteins

**DOI:** 10.1021/acsami.5c06927

**Published:** 2025-06-16

**Authors:** Masatoshi Maeki, Ayuka Niwa, Shota Oyama, Kyoko Aratani, Rina Ito, Yuichi Suzuki, Yusuke Sato, Akihiko Ishida, Hideyoshi Harashima, Manabu Tokeshi

**Affiliations:** † Division of Applied Chemistry, Faculty of Engineering, 12810 Hokkaido University, Kita 13 Nishi 8, Kita-ku, Sapporo 060-8628, Japan; ‡ Graduate School of Chemical Sciences and Engineering, Hokkaido University, Kita 13 Nishi 8, Kita-ku, Sapporo 060-8628, Japan; § Faculty of Engineering, Hokkaido University, Kita 13 Nishi 8, Kita-ku, Sapporo 060-8628, Japan; ∥ Faculty of Pharmaceutical Sciences, Hokkaido University, Kita 12 Nishi 8, Kita-ku, Sapporo 060-0812, Japan

**Keywords:** exosome, exosome-mimicking nanoparticles, mRNA delivery, drug delivery system, integrin, microfluidics

## Abstract

Exosomes, which are cell-secreted lipid-based nanoparticles, play a crucial role in intercellular communication by encapsulating and delivering various biomolecules such as DNA, mRNA, miRNA, and proteins. They offer potential as drug delivery systems (DDSs) based on their ability to cross biological barriers, use natural communication mechanisms, and minimize immunogenicity. However, the heterogeneity of exosomes presents a bottleneck for functional analysis and the development of exosome-based DDSs. Therefore, engineering techniques are needed to produce exosomes or exosome-mimicking nanoparticles with controlled characteristics, including the presentation of specific exosomal proteins on their surface. Here, a one-step microfluidic method for producing exosome-mimicking lipid-based nanoparticles decorated with specific exosomal proteins was developed, enabling control over the composition and characteristics of the resulting exosomes. Exosome-mimicking nanoparticles decorated with tetraspanin proteins (CD9, CD63, CD81) and integrins (ITG αVβ5, ITG α6β4), which are involved in cell signaling and organ targeting, were thereby generated. Investigating the impact of these exosomal proteins on RNA delivery efficiency revealed that ITG αVβ5-decorated exosome-mimicking nanoparticles significantly enhance RNA delivery both in vitro and in vivo. This study provides an approach for producing precisely decorated exosome-mimicking nanoparticles, which may be applied to elucidate the functions of exosomal proteins and develop targeted DDSs.

## Graphical abstract

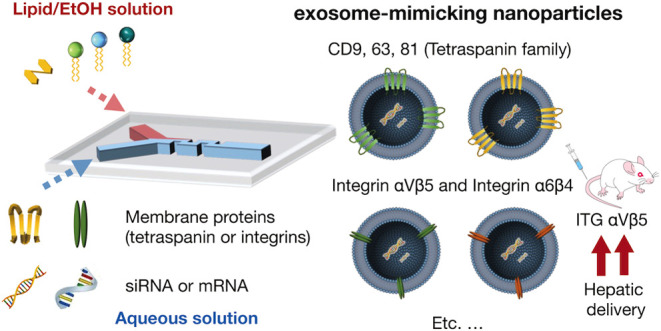

## Introduction

RNA delivery technologies are revolutionizing the treatment of infectious and intractable diseases. mRNA vaccines for COVID-19, which used lipid nanoparticles (LNPs) as carriers, have demonstrated high preventive efficacy.^1,2^ RNA-loaded nanoparticles are a promising technology for treating various diseases and preventing infectious diseases.^3^ In addition to LNPs, various lipid-based nanoparticles have been reported as RNA delivery carriers, including lipid-polymer hybrid nanoparticles,^4,5^ virus-like particles,^6,7^ and exosomes.^8–10^

Exosomes are cell-secreted extracellular vesicles, ranging from 30 to 200 nm in size, and are composed of a lipid bilayer membrane encapsulating various biomolecules such as DNA, mRNA, miRNA, and proteins.^11^ These naturally secreted nanoparticles circulate throughout the body and play a crucial role in intercellular communication and cancer metastasis. Notably, exosomes derived from mesenchymal stem cells have shown therapeutic potential in various disease models, suggesting their potential as an alternative to cell therapy. On the basis of their biological ability to carry and deliver RNA and proteins, exosomes are promising candidates for the development of targeted drug delivery systems (DDSs).^12–14^ Exosome-based DDSs offer numerous advantages. For example, exosomes can cross biological barriers, such as the blood–brain barrier, enabling targeted drug delivery to the brain.^15^ By using their natural mechanisms in intercellular communication and metastasis,^16,17^ exosomes can efficiently deliver drugs to recipient cells. Furthermore, autologous exosomes derived from the patient can minimize immunogenicity and reduce potential toxicity.^18^ These advantages demonstrate the potential of exosomes as safe and effective drug delivery carriers.

Exosomes are emerging as a new modality for nanomedicines; however, several challenges remain in their application in DDSs, including the need for multiple purification processes and a deeper understanding of the role of exosomal proteins in exosome functions.^14,19,20^ In particular, elucidating the roles of exosomal proteins, such as tetraspanins and integrins, is crucial for designing exosome-based nanomedicines. CD9, CD63, and CD81 are representative tetraspanins found on the exosome surface that may serve as markers for exosome detection in diagnostic applications. Integrins (ITGs) are also exosomal proteins, and ITG α6β4 and ITG αVβ5 on the exosome surface are associated with lung and liver metastasis;^21^ however, their role in cellular uptake and RNA delivery efficiency remains unclear. The particle size, lipid composition, and exosomal proteins on the exosome surface vary depending on the cell type. Moreover, even exosomes secreted from the same cell can exhibit heterogeneous characteristics. Therefore, the development of engineering techniques for the production of exosomes or exosome-mimicking nanoparticles that present specific exosomal proteins will enhance our understanding of the role of exosomal proteins in RNA delivery and the functions of cell-derived exosomes.

In this study, we developed a one-step microfluidic production method for exosome-mimicking lipid-based nanoparticles with the aim of understanding the role of exosomal proteins, in particular ITGs, in RNA delivery. The concept of the microfluidic production method for exosome-mimicking particles is shown in Figure 1. Recently, microfluidic devices have been employed for RNA-loaded LNP production.^12,22^ Microfluidic devices enable the production of precisely size-controlled RNA-loaded LNPs with good reproducibility by rapid mixing of a lipid/ethanol solution and an RNA/aqueous solution.^23–25^ We hypothesized that exosome-mimicking nanoparticles decorated with exosomal proteins could be produced by adding proteins into the RNA/aqueous solution. We expected that, during the self-assembly process driven by ethanol dilution in the microchannel, the exosomal proteins would be incorporated into the lipid membrane without denaturation, aided by microfluidic rapid mixing. We demonstrated the production of siRNA- or mRNA-loaded exosome-mimicking nanoparticles decorated with tetraspanins (CD9, CD63, or CD81) and ITGs (αVβ5 or α6β4), using the microfluidic device. In addition, the ITG αVβ5-decorated exosome-mimicking nanoparticles enhanced RNA delivery efficiency both in vitro and in vivo.

**Figure 1:**
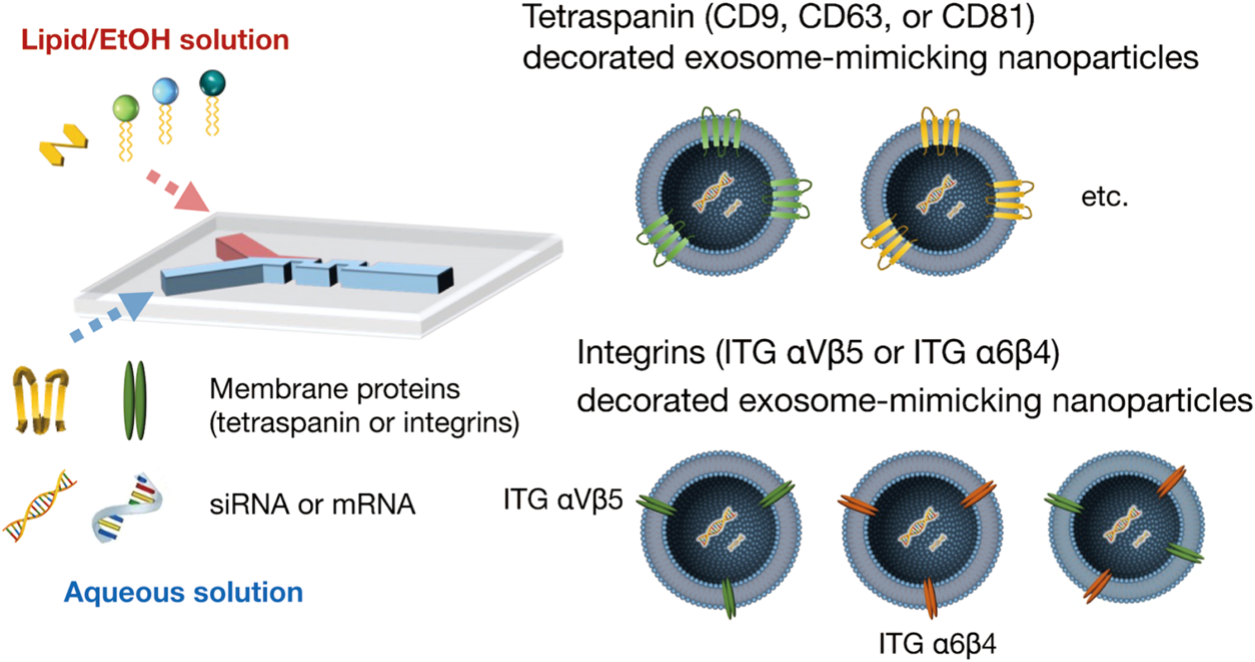
Concept of the one-step production of exosome-mimicking nanoparticles using a microfluidic device. The exosome-mimicking lipid nanoparticle surface was decorated with tetraspanins (CD9, CD63, or CD81) or integrins (ITG αVβ5 or ITG α6β4) during the self-assembly process of nanoparticles.

## Results and Discussion

### One-Step Production of Exosome-Mimicking Nanoparticles: A Proof-of-Concept Study

One advantage of microfluidic-based LNP production is the rapid dilution of ethanol, which allows for the generation of LNPs with a uniform size distribution.^23^ Rapid ethanol dilution induces the self-assembly of lipid molecules. In this study, we decorated the surface of the nanoparticles with exosomal proteins, including tetraspanins (CD9, CD63, and CD81) and ITGs (ITG αVβ5 and ITG α6β4). We hypothesized that these exosomal proteins would incorporate into the surface of the LNPs during the self-assembly process. To mimic exosome characteristics such as lipid composition, negative surface charge, and size, we used a five-lipid system that included phosphocholine, sphingomyelin, phosphoethanolamine, cholesterol, and phosphatidylserine.^26^ The molar ratio of these lipids was DOPC/sphingomyelin/DOPE/cholesterol/DOPS at 28/17.5/17.5/30/7. This lipid mixture dissolved in ethanol was diluted with Tris-Cl buffer containing siRNA and exosomal proteins. First, we conducted a proof-of-concept study and examined the effect of the flow rate ratio (FRR: the ratio of the aqueous phase to the ethanol phase) on the presentation efficiency of CD63 (Figure S1). The protein weight to lipid molar ratio was fixed at 0.8 g/mol among different FRR conditions after mixing. As a result, we confirmed that the microfluidic approach was able to produce exosome-mimicking nanoparticles, which were decorated with CD63 on their surface without denaturation by ethanol. We expected that the high FRR conditions could decorate CD63 on the nanoparticle surface because ethanol was rapidly diluted under the high FRR conditions, preventing the denaturation of CD63. However, contrary to our expectations, a FRR of 2 produced the highest CD63 decorated exosome-mimicking nanoparticles, and this was therefore considered the optimized flow condition.

Figure 2 shows the characteristics of exosome-mimicking nanoparticles decorated with CD9, CD63, or CD81. We explored various protein weight-to-lipid molar (Pw/Lm) ratios for CD9, CD63, and CD81 and found that the amount of surface protein on the exosome-mimicking nanoparticles increased with increasing protein in the Pw/Lm ratio. When the Pw/Lm ratios were 2.2, 4.0, and 2.4 g/mol for CD9, CD63, and CD81, respectively, the corresponding amounts of surface-presented proteins were 235, 3934, and 3720 ng/mL. The size of each exosome-mimicking nanoparticle was approximately 100 nm, with a polydispersity index (PDI) of 0.2, regardless of the protein type. The ζ-potential was −20 to −40 mV, which was similar to that of cell-derived exosomes. We also evaluated the siRNA encapsulation efficiency of each exosome-mimicking nanoparticle under this preparation condition (Figure S2). The exosome-mimicking nanoparticles showed an siRNA encapsulation efficiency of 50 to 60%. Although the siRNA encapsulation efficiency of the CD63-decorated exosome-mimicking nanoparticles was similar to that of CD63(−) particles, CD9- and CD81-decorated exosome-mimicking nanoparticles showed reduced siRNA encapsulation efficiency compared with CD9(−) and CD81(−) particles, respectively. Interestingly, the amount of CD9 presented on the exosome-mimicking nanoparticles prepared by the microfluidic device was 10 times higher than that of the batch method (Figure S3). We confirmed that the presence of CD9 did not affect the inner structure of the exosome-mimicking nanoparticles by small-angle X-ray scattering (Figure S4).^27,28^ These results indicated that the preparation method, FRR, and Pw/Lm ratio play a crucial role in the properties of exosome-mimicking nanoparticles.

**Figure 2:**
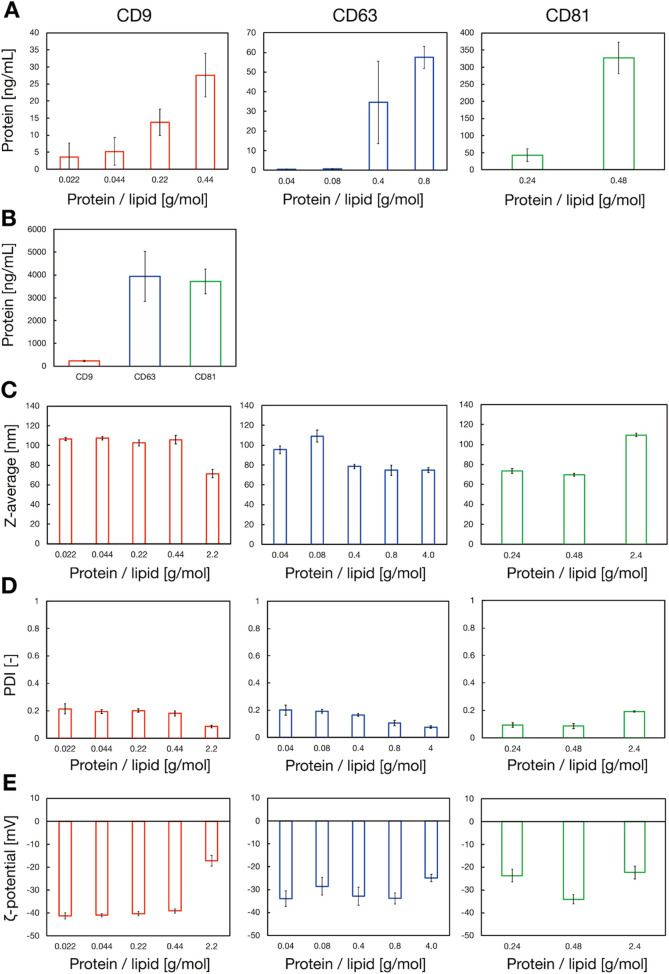
Characterization of exosome-mimicking nanoparticles decorated with CD9, CD63, or CD81 at different protein/lipid molar ratios. (A) Presentation efficiency relative to the protein/lipid molar ratio. (B) Amount of each protein presented on the surface. Exosome-mimicking nanoparticles were produced at protein/lipid molar ratios of 2.2, 4.0, and 2.4 g/mol for CD9, CD63, and CD81, respectively. (C–E) Average size (C), polydispersity index (PDI) (D), and ζ-potential (E) of the exosome-mimicking nanoparticles. The results of three replicate measurements are shown together with their average and standard deviation (mean ± SD, *n* = 3).

Next, we applied this microfluidic production method to the production of ITG-decorated exosome-mimicking nanoparticles. ITG αVβ5 and ITG α6β4 were chosen as model proteins because these proteins expressed on the exosome surface play an important role in delivering exosomes to the liver and lungs.^21^ We optimized the Pw/Lm ratio to 4.0 and 3.6 g/mol for ITG αVβ5 and ITG α6β4, respectively. The presentation of ITGs on the exosome-mimicking nanoparticles was determined using antibody-immobilized magnetic beads and flow cytometry (FCM) (Figure 3A,B). DiD-labeled ITG αVβ5- or ITG α6β4-decorated exosome-mimicking nanoparticles were measured by FCM after size-exclusion chromatography to remove the free proteins. From the FCM measurement, we confirmed the presence of ITGs on the exosome-mimicking nanoparticle surface. The size, PDI, and ζ-potential of the exosome-mimicking nanoparticles were 120 nm, 0.2, and −40 mV, respectively, irrespective of the ITG type. However, the siRNA encapsulation efficiency was dramatically decreased compared with that of tetraspanin-decorated exosome-mimicking nanoparticles. In this study, we used the extracellular domains of CD9, CD63, and CD81, which have molecular weights ranging from 11 to 23 kDa, to dissolve the aqueous solution because these proteins are four-pass transmembrane proteins. By contrast, the molecular weights of ITG αVβ5 and ITG α6β4 were 200 and 190 kDa, respectively. Therefore, the difference in molecular weight may affect the siRNA encapsulation efficiency. These results demonstrate that our microfluidic approach can produce exosome-mimicking nanoparticles decorated with tetraspanins or ITGs. The exosome-mimicking nanoparticles produced in this study have the potential to be used as tools for investigating exosome function and as standard exosome particles for developing new exosome detection methods. However, the low siRNA encapsulation efficiency needs to be improved for DDS applications and for understanding exosome functions.

**Figure 3:**
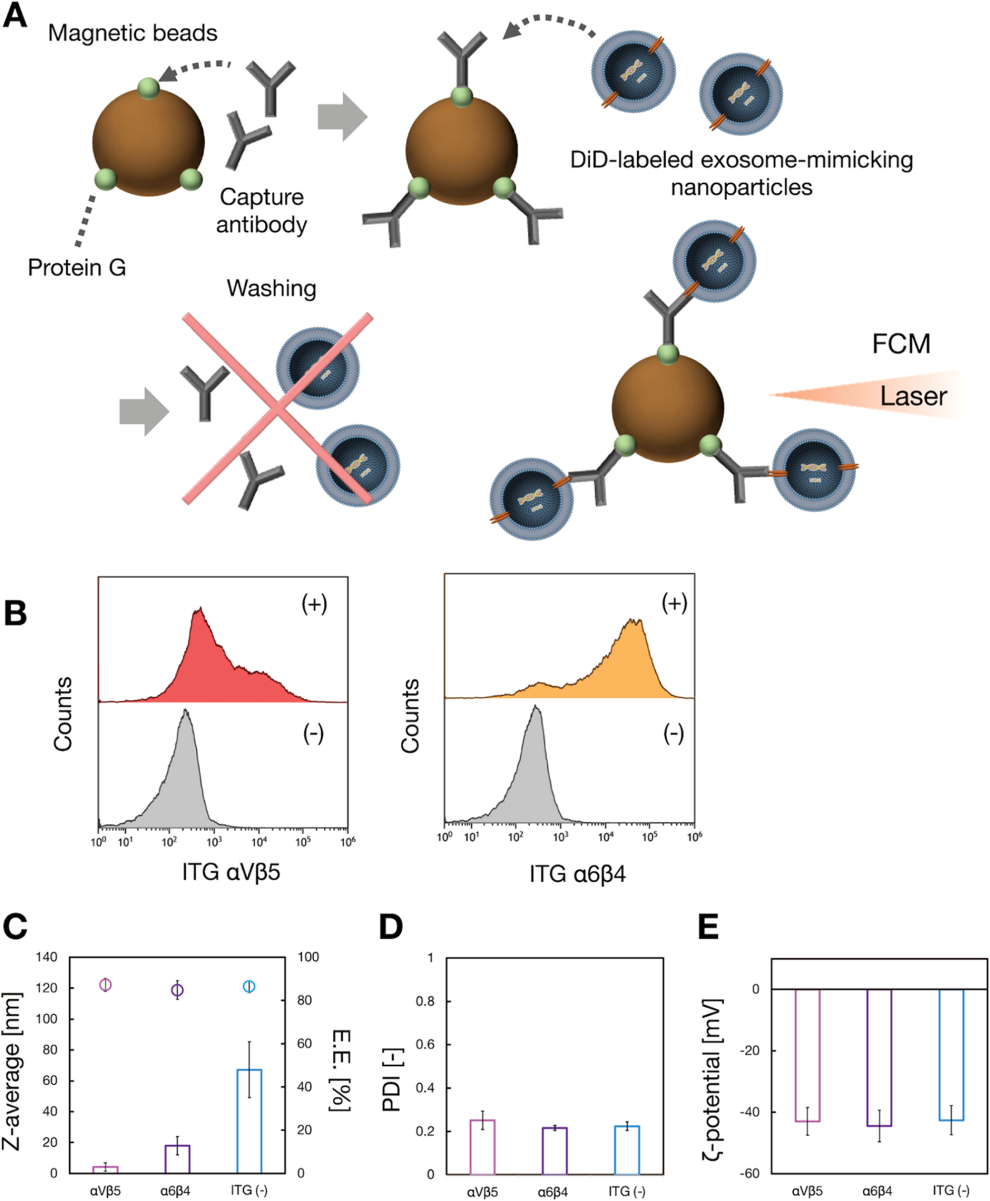
Characterization of integrin-decorated exosome-mimicking nanoparticles. (A) Schematic illustration of ITG-decorated exosome-mimicking nanoparticle measurement using antibody-immobilized magnetic beads and flow cytometry. Magnetic beads were conjugated with antibodies against ITG αVβ5 or ITG α6β4, followed by the addition of DiD-labeled exosome-mimicking nanoparticles. The Pw/Lm ratios were set at 4.0 and 3.6 g/mol for ITG αVβ5 and ITG α6β4. The FRR was 2. Free antibodies and exosome-mimicking nanoparticles without ITG presentation were removed by washing. The fluorescence intensity of the beads was measured using a CytoFLEX flow cytometer (excitation and emission wavelengths: 638 and 660 nm, respectively). (B) Measurement results of ITG αVβ5- or ITG α6β4-decorated exosome-mimicking nanoparticles. The fluorescence profiles of ITG (−) nanoparticles are indicated by (−). (C–E) Average particle size (bar graph) and siRNA encapsulation efficiency (E.E., open circle) (C), PDI (D), and ζ-potential (E) of ITG αVβ5- or ITG α6β4-decorated exosome-mimicking nanoparticles and ITG (−). The results of three replicate measurements are shown together with their average and standard deviation (mean ± SD, *n* = 3).

### Characterization of Exosome-Mimicking Nanoparticles Composed of Ionizable Lipids

To improve the siRNA encapsulation efficiency, an ionizable lipid, SM-102, was added to the lipid mixture. For achieving both high siRNA encapsulation efficiency and exosome-like characteristics (negative surface charge and presentation of exosomal proteins), the lipid composition was optimized while minimizing the amount of SM-102. We also explored different helper lipids and replaced DOPC with DSPC. The optimized molar ratio of the lipid mixture was SM-102/DSPC/sphingomyelin/DOPE/cholesterol/DOPS/DMG-PEG 2k (20/11/20/5/30/7/1.5). After the lipid composition optimization, ITG αVβ5- or ITG α6β4-decorated exosome-mimicking particles were produced in the same manner as previously described. Figures 4 and S5 show the characteristics of exosome-mimicking particles prepared with a lipid composition containing ionizable lipids. With this lipid composition, the size, PDI, and ζ-potential of the exosome-mimicking nanoparticles were 120 nm, 0.13 to 0.19, and −7 to 8 mV, respectively, irrespective of the ITG type. Notably, the siRNA encapsulation efficiency was 90%. In addition, we confirmed the presentation of ITGs on the exosome-mimicking nanoparticle surface by FCM. Addition of the ionizable lipid improved the siRNA encapsulation efficiency, while the nanoparticles maintained exosome characteristics, including a negative charge and the presence of exosomal proteins.

**Figure 4:**
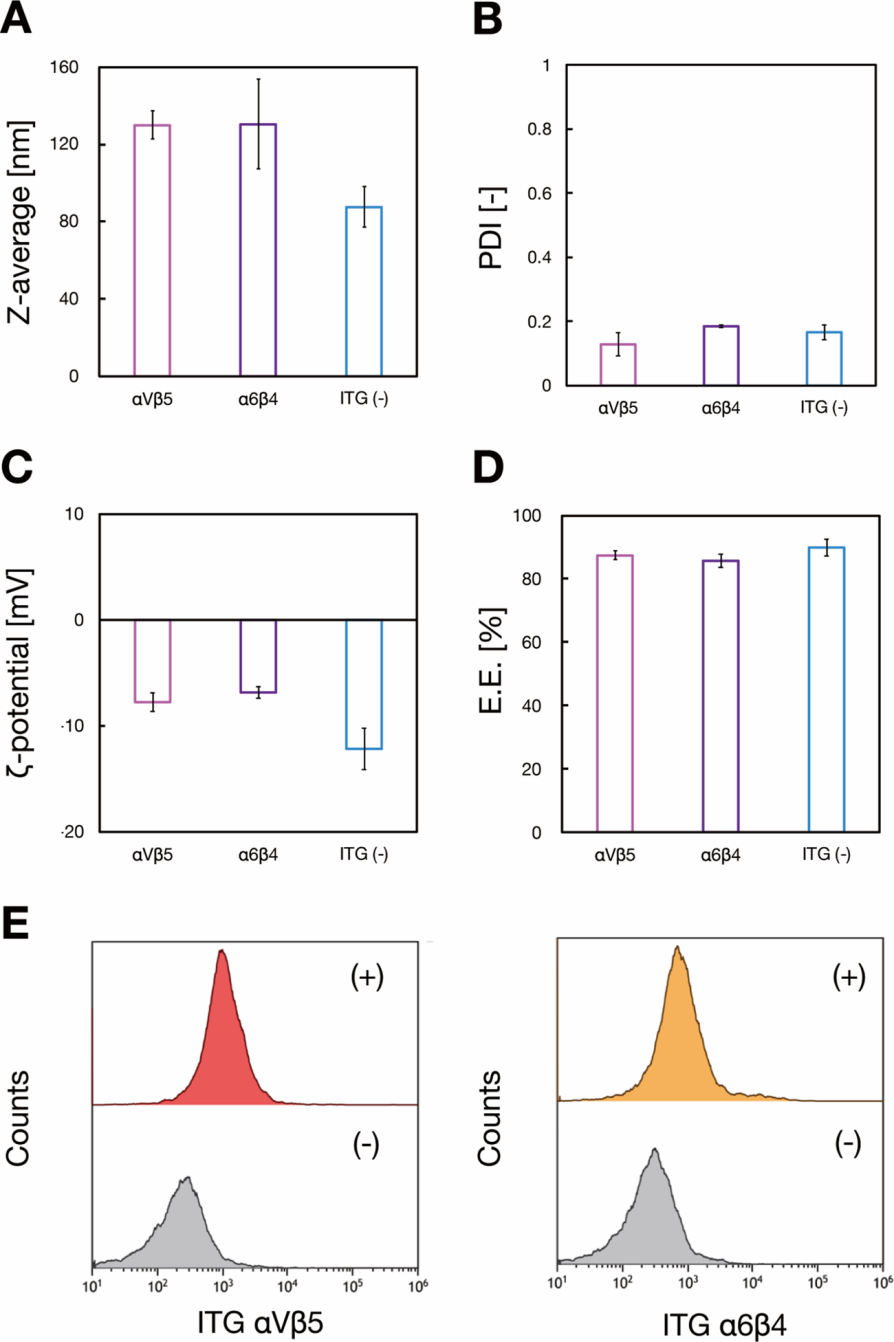
Characterization of siRNA-loaded integrin-decorated exosome-mimicking nanoparticles composed of ionizable lipids. (A–D) Average particle size (A), PDI (B), ζ-potential (C), and siRNA encapsulation efficiency (E.E.) (D) of ITG αVβ5- or ITG α6β4-decorated exosome-mimicking nanoparticles and ITG (−) particles. (E) Measurement results of ITG αVβ5- or ITG α6β4-decorated exosome-mimicking nanoparticles using antibody-immobilized magnetic beads and FCM. The fluorescence profiles of ITG (−) nanoparticles are indicated by (−). The fluorescence intensity of the beads was measured using a CytoFLEX flow cytometer (excitation and emission wavelengths: 638 and 660 nm, respectively). The ITG weight to lipid molar ratios were set at 4.0 and 3.6 g/mol for ITG αVβ5 and ITG α6β4, respectively. The results of three replicate measurements are shown together with their average and standard deviation (mean ± SD, *n* = 3).

Then, we evaluated the gene silencing performance of ITG-decorated exosome-mimicking nanoparticles and investigated the role of ITGs in RNA delivery. Figure 5A,B present the luciferase knockdown activity and cell viability following treatment with exosome-mimicking nanoparticles or ITG (−) particles. As shown in Figure 5A, both ITG-decorated exosome-mimicking nanoparticles showed higher luciferase knockdown activity than the particles without ITG. At a 60 nM dosage, the ITG αVβ5-decorated exosome-mimicking nanoparticles, ITG α6β4-decorated exosome-mimicking nanoparticles, and ITG (−) particles exhibited 52, 45, and 34% luciferase knockdown efficiency, respectively. Figure 5C,D illustrates the luciferase knockdown performance and cytotoxicity of dual-protein-decorated and tetraspanin-decorated exosome-mimicking nanoparticles. Interestingly, dual decoration with ITG αVβ5 and ITG α6β4 enhanced the luciferase knockdown performance to 75%. However, in comparison with ITG-decorated exosome-mimicking nanoparticles, tetraspanin-decorated exosome-mimicking nanoparticles did not enhance the luciferase knockdown performance, even with dual tetraspanin decoration using CD9 and CD81. Then, we prepared four types of exosome-mimicking nanoparticles dual-decorated with ITG and tetraspanin protein. The dual decoration by ITGs and tetraspanins also enhanced the luciferase knockdown performance, similar to the ITG dual decorated exosome-mimicking nanoparticles, in all cases. From these results, we found that ITGs, in particular ITG αVβ5, play a crucial role in RNA delivery. In addition, codecoration with ITG αVβ5 and α6β4 or with ITGs and tetraspanins enhanced RNA delivery performance. The main functions of ITGs are as receptors for cell adhesion to the extracellular matrix and for intercellular signaling. However, cell-derived exosomes are highly heterogeneous, and present multiple exosomal proteins such as tetraspanins (CD9, CD63, and CD81), ITGs, Major Histocompatibility Complexs, and glycoproteins. Therefore, understanding the role of each exosomal protein in RNA delivery performance is difficult using cell-derived exosomes. By contrast, exosome-mimicking nanoparticles, which are decorated with desired exosomal proteins, allow us to understand their detailed functions in RNA delivery. Our results suggest that the protein profile on the exosome surface affects the cellular uptake pathway and RNA delivery performance.

**Figure 5:**
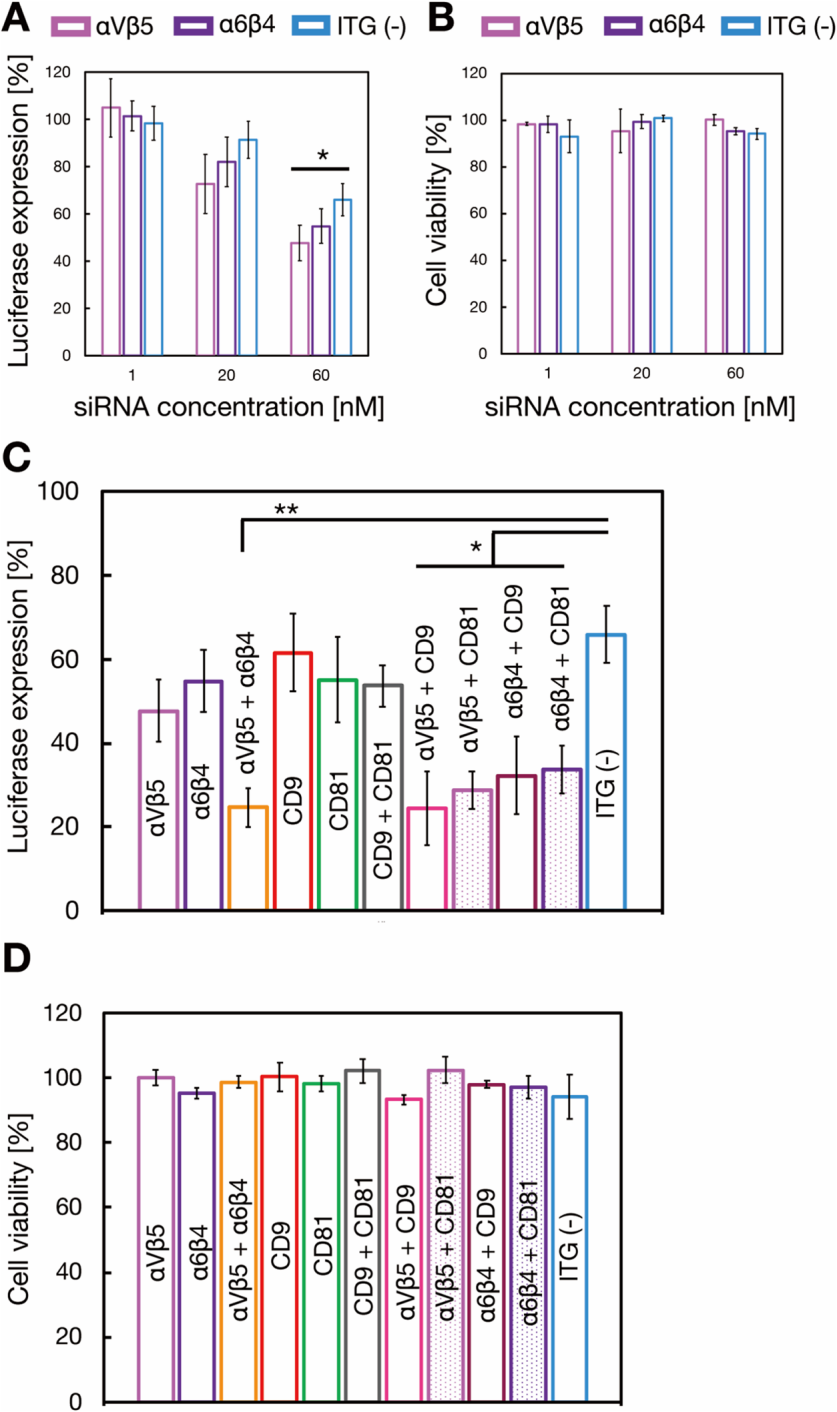
Luciferase knockdown performance and cell viability of exosome-mimicking nanoparticles. (A, B) Luciferase knockdown performance (A) and cell viability (B) of ITG αVβ5- or ITG α6β4-decorated exosome-mimicking nanoparticles or ITG (−) particles at doses of 1, 20, and 60 nM siRNA. (C, D) Luciferase knockdown performance (C) and cell viability (D) of codecorated exosome-mimicking nanoparticles at a dose of 60 nM siRNA. The weight-to-molar ratios of tetraspanin protein to lipid were set to 0.44 and 0.48 g/mol for CD9 and CD81, respectively. The results of three replicate measurements are shown together with their average and standard deviation (mean ± SD, *n* = 3). *p < 0.05 and **p < 0.01.

### Production and Characterization of mRNA-Loaded ITG-Decorated Exosome-Mimicking Nanoparticles

To understand the role of exosomal proteins in RNA delivery in vivo, we produced mRNA-loaded ITG-decorated exosome-mimicking nanoparticles. Compared with siRNA-loaded exosome-mimicking nanoparticles, mRNA-loaded exosome-mimicking nanoparticles are preferable for in vivo studies because they enable the direct measurement of protein expression levels. Using these mRNA-loaded exosome-mimicking nanoparticles, we investigated the cellular uptake pathway in vitro and evaluated the biodistribution and luciferase expression in vivo. Figure 6A shows the characteristics of the mRNA-loaded ITG-decorated exosome-mimicking nanoparticles. In comparison to the siRNA-loaded exosome-mimicking nanoparticles, the size of the mRNA-loaded exosome-mimicking nanoparticles was slightly different depending on the ITG type. The size of the mRNA-loaded exosome-mimicking nanoparticles was 143 and 105 nm for ITG αVβ5 and ITG α6β4, respectively. The ζ-potential of the exosome-mimicking nanoparticles was −3 mV, similar to that of the ITG (−) nanoparticles. The mRNA encapsulation efficiency of the ITG-decorated exosome-mimicking nanoparticles was 70 to 80%. In addition, FCM measurement using antibody-immobilized magnetic beads indicated that ITG αVβ5 and ITG α6β4 were presented on the nanoparticle surface. These findings demonstrated that our one-step microfluidic production method can be applied to mRNA-loaded exosome-mimicking nanoparticles.

**Figure 6:**
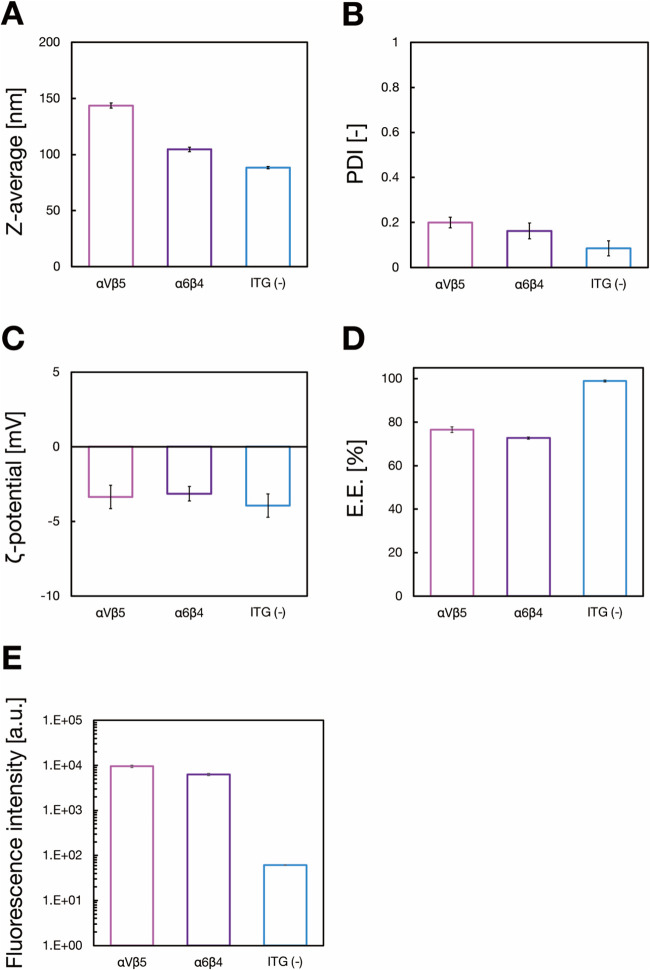
Characterization of mRNA-loaded integrin-decorated exosome-mimicking nanoparticles composed of ionizable lipids. (A–D) Average particle size (A), PDI (B), ζ-potential (C), and mRNA encapsulation efficiency (E.E.) (D) of ITG αVβ5- or ITG α6β4-decorated exosome-mimicking nanoparticles and ITG (−) particles. (E) Quantitative measurements of the ITG presentation on the exosome-mimicking nanoparticles using antibody-immobilized magnetic beads and FCM (excitation and emission wavelengths: 638 and 660 nm, respectively). The ITG weight to lipid molar ratios were set at 4.0 and 3.6 g/mol for ITG αVβ5 and ITG α6β4, respectively. The results of three replicate measurements are shown together with their average and standard deviation (mean ± SD, *n* = 3).

Then, we evaluated the luciferase expression performance and cell viability of the ITG-decorated exosome-mimicking nanoparticles (Figure 7). Similar to the siRNA-loaded exosome-mimicking nanoparticles, the ITG αVβ5-decorated exosome-mimicking nanoparticles exhibited the highest luciferase expression performance. The ITG α6β4-decorated exosome-mimicking nanoparticles also showed higher luciferase expression performance than the ITG (−) nanoparticles. The luciferase expression level of the ITG αVβ5-decorated exosome-mimicking nanoparticles was 3.4- to 4.9-fold higher than that of the ITG (−) particles, indicating that the presentation of ITG enhances mRNA delivery performance.

**Figure 7:**
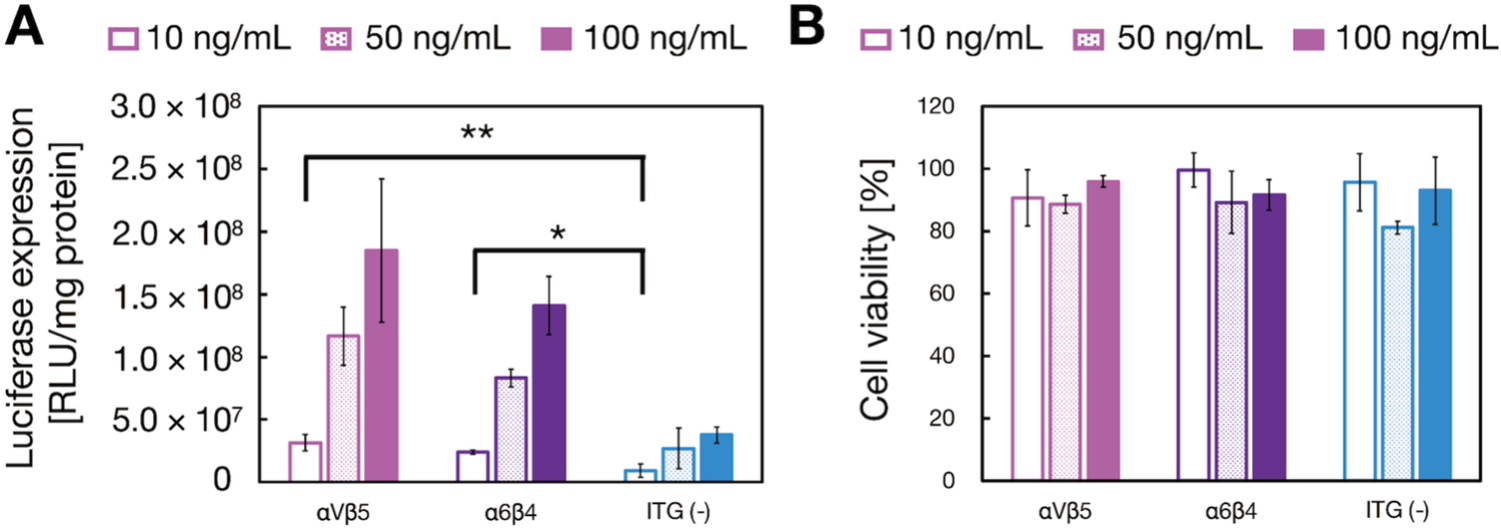
(A) Luciferase expression performance and (B) cell viability of mRNA-loaded ITG-decorated exosome-mimicking nanoparticles at doses of 10, 50, and 100 ng/mL mRNA/well. The results of three replicate measurements are shown together with their average and standard deviation (mean ± SD, *n* = 3). *p < 0.05 and **p < 0.01. N. S.: not significant.

To elucidate the RNA delivery mechanism of the exosome-mimicking nanoparticles, we performed an endocytosis inhibition assay. Chlorpromazine (CPZ), genistein (Gen), 5-(*N*-ethyl-*N*-isopropyl) amiloride (EIPA), and dynasore (Dyn) were used as endocytosis inhibitors for clathrin-mediated endocytosis, caveolae-mediated endocytosis, macropinocytosis, and dynamin-dependent endocytosis, respectively.^29,30^ The cellular uptake of all of the exosome-mimicking nanoparticles was decreased in the presence of all of the inhibitors (Figure 8). These results indicated that the exosome-mimicking nanoparticles were internalized into cells via multiple pathways. Notably, the cellular uptake of ITG αVβ5-decorated exosome-mimicking nanoparticles was lower than that of ITG (−) particles in the presence of CPZ and EIPA, suggesting that ITG αVβ5 enhances mRNA delivery efficiency via clathrin-mediated endocytosis and macropinocytosis. As shown in Figure 6A, the ITG αVβ5-decorated exosome-mimicking nanoparticles were 143 nm in size, whereas the ITG α6β4-decorated exosome-mimicking nanoparticles and ITG (−) particles were 105 and 88 nm in size, respectively. The upper size limit for internalization by clathrin-mediated endocytosis is approximately 200 nm.^31^ In addition, macropinocytosis has been reported to be a highly efficient pathway for RNA delivery.^32^ Therefore, ITG αVβ5 may enhance mRNA delivery efficiency via clathrin-mediated endocytosis and macropinocytosis. We also evaluated the luciferase expression performance in the presence of the inhibitors. The luciferase expression level was decreased in the presence of CPZ and EIPA, whereas it was increased in the presence of Gen. These results suggest that the inhibition of the caveolae-dependent pathway by Gen may promote the uptake of exosome-mimicking nanoparticles via other pathways, leading to increased luciferase expression. In other words, exosome-mimicking nanoparticles internalized via the caveolae-dependent pathway may contribute less to RNA delivery. We revealed that the clathrin-dependent pathway and macropinocytosis are the significant internalization pathways for RNA delivery by ITG αVβ5-decorated exosome-mimicking nanoparticles.

**Figure 8:**
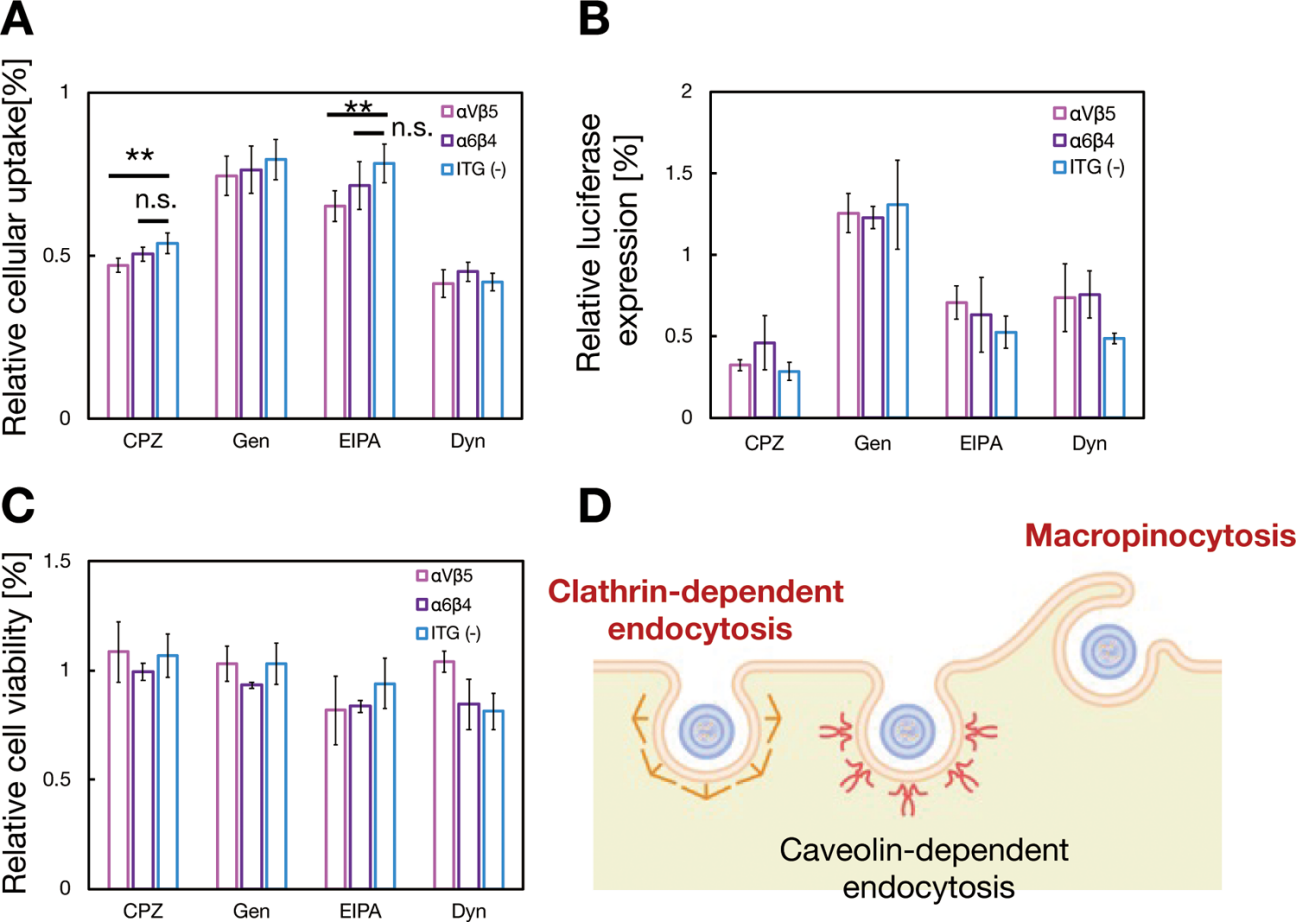
Endocytosis inhibition assay using mRNA-loaded ITG-decorated exosome-mimicking nanoparticles. (A–C) Relative cellular uptake (A), luciferase expression efficiency (B), and cell viability (C). (D) Schematic illustration of the endocytosis pathway of ITG-decorated exosomes. Chlorpromazine (CPZ), genistein (Gen), 5-(*N*-ethyl-*N*-isopropyl) amiloride (EIPA), and dynasore (Dyn) were used as endocytosis inhibitors. The mRNA dose was 100 ng/mL. Each relative value was normalized to the corresponding result without endocytosis inhibitor. The results of three replicate measurements are shown together with their average and standard deviation (mean ± SD, *n* = 3). **p < 0.01. N.S.: not significant.

### In Vivo Evaluation of mRNA-Loaded Exosome-Mimicking Nanoparticles

To demonstrate the potential of exosome-mimicking nanoparticles for in vivo mRNA delivery, we evaluated the biodistribution and luciferase expression of ITG-decorated exosome-mimicking nanoparticles in mice. Hoshino et al. reported that ITG αVβ5 and ITG α6β4 on exosomes promote liver and lung metastasis, respectively.^21^ ITGs have been reported to mediate cancer progression, metastasis, and cell migration.^33,34^ Thus, we expected that the biodistribution of ITG-decorated exosome-mimicking nanoparticles would be similar to that of natural exosomes. However, the detailed roles of exosomes in RNA delivery remain unclear. Therefore, we investigated the role of exosomes in RNA delivery by measuring the luciferase expression level following the delivery of mRNA using ITG-decorated exosome-mimicking nanoparticles.

Figure 9A,B show the biodistribution of DiR-labeled mRNA-loaded ITG-decorated exosome-mimicking nanoparticles. We confirmed the accumulation of ITG-decorated exosome-mimicking nanoparticles and ITG (−) particles in the liver and spleen. The presence of ITGs on the surface did not affect the biodistribution. However, the luciferase expression profile was affected by the presence of ITGs (Figure 9B,C). The luciferase expression level in the liver was 5.2-fold and 2.2-fold higher for the ITG αVβ5-decorated exosome-mimicking nanoparticles than for the ITG α6β4-decorated exosome-mimicking nanoparticles and ITG (−) particles, respectively. We also observed luciferase expression in the spleen with all particles. These results indicate that ITG αVβ5 plays a role in promoting protein expression in the liver, although it did not enhance accumulation in the liver. We consider that the higher luciferase expression shown by ITG αVβ5-decorated exosome-mimicking nanoparticles may be attributed to their enhanced delivery to hepatocytes compared to both nanoparticles decorated with ITG α6β4 and those without ITG decoration. In contrast to the ITG αVβ5-decorated exosome-mimicking nanoparticles, the ITG α6β4-decorated exosome-mimicking nanoparticles showed lower luciferase expression levels than the ITG (−) particles in both the liver and spleen. Additionally, ITG α6β4-decorated exosome-mimicking nanoparticles did not exhibit lung accumulation similar to that observed with natural exosomes. Previous research has shown that cell origins of exosomes affect their biodistribution.^21^ In this study, we used recombinant ITGs expressed by HEK 293 cells; therefore, the biodistribution might differ depending on the host organisms or cell lines used to produce the recombinant proteins.

**Figure 9:**
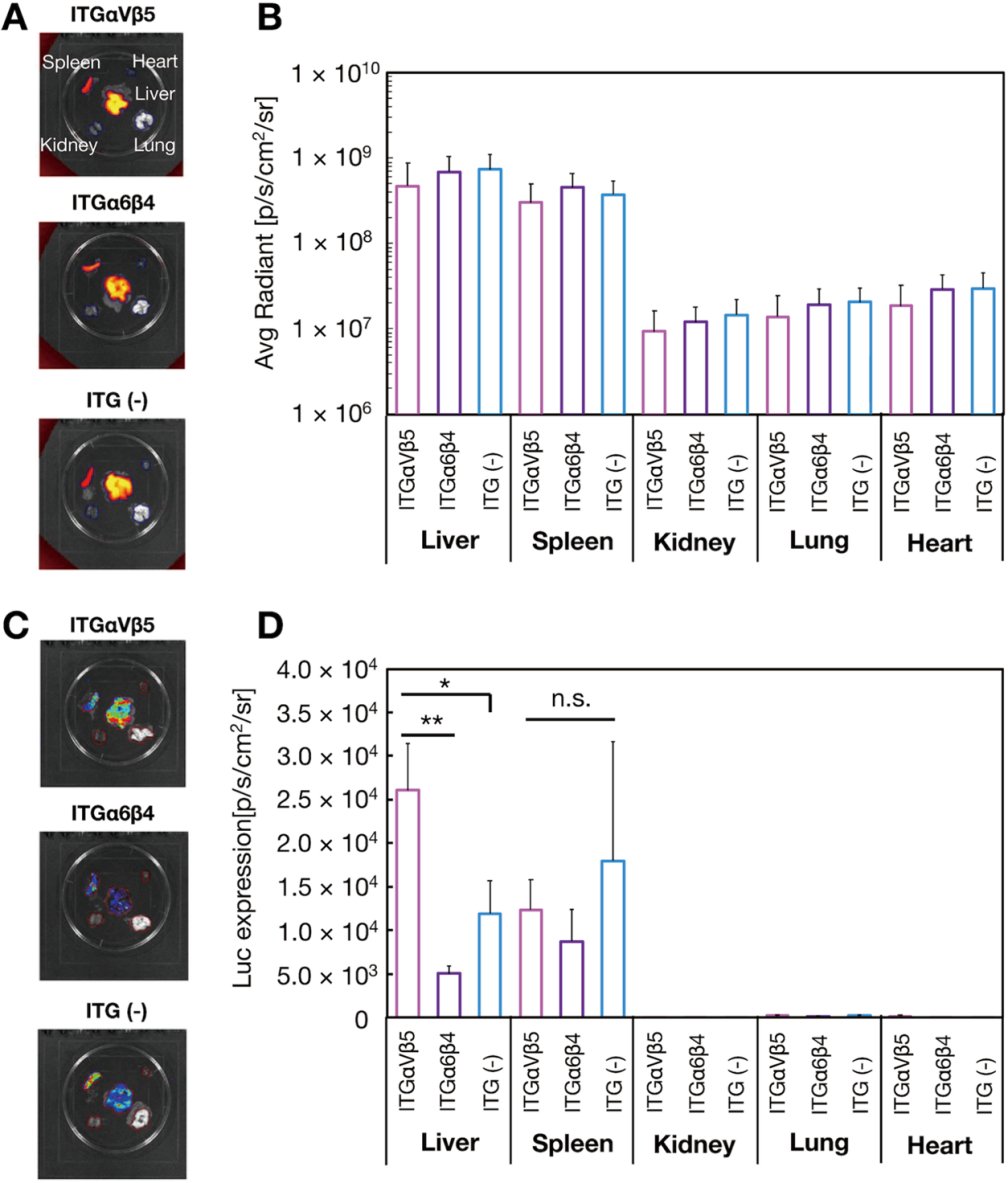
Biodistribution and luciferase expression in vivo. (A) Biodistribution of DiR-labeled ITG αVβ5- or ITG α6β4-decorated exosomes, and ITG (−) nanoparticles. (B) Quantitative analysis of biodistribution. Ex vivo imaging was performed 3 h postadministration. (C) Luciferase expression of ITG αVβ5- or ITG α6β4-decorated exosomes, and ITG (−) nanoparticles. (D) Quantitative analysis of luciferase expression. Mice were injected via the tail vein with 200 μL of mRNA-loaded exosome-mimicking nanoparticles or mRNA-loaded LNPs (0.05 mg mRNA/kg) for intravenous injection. Ex vivo imaging was performed 6 h postadministration. The results of three replicate measurements are shown together with their average and standard deviation (mean ± SD, *n* = 3). *p < 0.05 and **p < 0.01. N.S.: not significant.

Further research is needed to investigate the specific effects of the ionizable lipid on the biodistribution of exosome-mimicking nanoparticles. In this study, we used an ionizable lipid to efficiently encapsulate mRNA. However, because the ionizable lipid is not present in natural exosomes, it may have affected the biodistribution. Additionally, differences in lipid composition between natural exosomes and exosome-mimicking nanoparticles may affect serum protein adsorption in the blood.^35–38^ The formation of a protein corona on LNPs has been shown to influence their biodistribution.^39^ In addition to displaying tetraspanins and ITGs, exosomes, unlike LNPs, form protein coronas. Although the ITG αVβ5-decorated exosome-mimicking nanoparticles produced in this study did not show the increased liver accumulation reported for natural exosomes, they exhibited increased protein expression efficiency in the liver. Optimizing the combination and quantity of proteins displayed, as well as understanding and controlling protein corona formation, will be crucial for future applications of exosome-mimicking nanoparticles in DDSs and for elucidating the functions of natural exosomes.

## Conclusions

In this study, we demonstrated the one-step production of exosome-mimicking nanoparticles decorated with exosomal proteins using the microfluidic device. Tetraspanins, including CD9-, CD63-, and CD81-decorated exosome-mimicking nanoparticles, and ITG αVβ5- and ITG α6β4-decorated exosome-mimicking nanoparticles encapsulating siRNA or mRNA were produced without any complicated procedures such as protein expression, purification, or separation. We confirmed that ITGs played an important role in RNA delivery and dual decoration by ITG and tetraspanins, and two types of ITGs enhanced the luciferase knockdown performance. To our knowledge, this study is the first to report on the effect of ITGs on RNA delivery using exosome-mimic nanoparticles. In addition, we investigated the in vivo biodistribution and protein expression of the delivered mRNA and found that ITG αVβ5 also enhanced protein expression in the liver. The heterogeneity of cell-derived exosomes makes them difficult to use in diagnostics, DDSs, and research to understand their functions. This microfluidic-based approach enables the production of exosome-mimicking nanoparticles decorated with specific types and quantities of exosomal proteins, offering the potential for use as standardized particles in exosome research. Further improvements are needed to fully mimic the characteristics of cell-derived exosomes, including their biodistribution. Proteomic and lipidomic analyses of cell- and patient-derived exosomes can provide valuable information for refining the design and engineering of exosomes. This microfluidic-based production method has the potential to become a powerful technique for exosome-related research and advance the development of exosome-based DDSs.

## Experimental Section

### Materials

1,2-distearoyl-*sn*-glycero-3-phosphocholine (DSPC), 1,2-dioleoyl-*sn*-glycero-3-phosphocholine (DOPC), 1,2-dioleoyl-*sn*-glycero-3-phosphoethanolamine (DOPE), 1,2-dioleoyl-*sn*-glycero-3-phospho-l-serine (DOPS), and 1,2-dimyristoyl-rac-glycero-3-methoxypolyethylene glycol-2000 (DMG-PEG 2k) were purchased from NOF Corporation (Tokyo, Japan). Sphingomyelin from porcine brain was obtained from Avanti Polar Lipids (Alabaster, AL). siGL4 was synthesized by Hokkaido System Science Co., Ltd. (Sapporo, Japan). The siGL4 sequences for the sense and antisense strands were reported previously.^40^ Firefly luciferase-encoding mRNA was purchased from Trilink Bio-Technologies (San Diego, CA). Cholesterol, DMEM, and MEM were purchased form Sigma-Aldrich (St. Louis, MO). Heptadecan-9-yl 8-((2-hydroxyethyl)­(6-oxo-6-(undecyloxy)­hexyl)­amino)­octanoate (SM-102) was purchased from BLDpharm (Shanghai, China). Ethanol, methanol, sodium acetate, acetic acid, tris­(hydroxymethyl)­aminomethane, hydrochloride, 2-[4-(2-hydroxyethyl)-1-piperazinyl]­ethanesulfonic acid, D-PBS (−), and Cell Counting kit-8 were obtained from Fujifilm Wako Pure Chemical Corporation (Osaka, Japan). Fetal bovine serum (FBS), penicillin–streptomycin, trypsin (2.5%), Quant-iT RiboGreen RNA Reagent, BCA protein assay kit, DiD, and DiR were obtained from Thermo Fisher Scientific (Waltham, MA). G418 and Triton X-100 were purchased from Nacalai Tesque (Kyoto, Japan). The Dual-Glo Luciferase Assay System and ONE-Glo Luciferase Assay System were obtained from Promega (Madison, WI). CD9, CD63, and CD81 were purchased from Sino Biological (Beijing, China). Integrin (ITG) αVβ5, ITG α6β4, antihuman ITG αV antibody, and antihuman ITG α6 antibody were obtained from R&D Systems, Inc. (Minneapolis, MN). Chlorpromazine hydrochloride (CPZ), genistein (Gen), and 5-(*N*-ethyl-*N*-isopropyl) amiloride (EPIA) were purchased from Fujifilm Wako Pure Chemical Corporation. Dynasore (Dyn) was obtained from Tokyo Chemical Industry Co., Ltd. (Tokyo, Japan).

### Proof of Concept Experiment for the Exosome-Mimicking Nanoparticles Preparation

DOPC, sphingomyelin, DOPE, and cholesterol were dissolved in ethanol, and DOPS was dissolved in methanol to prepare the stock solutions. Lipid solutions were prepared by mixing each stock solution at molar ratios of DOPC/sphingomyelin/DOPE/cholesterol/DOPS of 28/17.5/17.5/30/7 and the total lipid concentration was adjusted to 10 mM. For the aqueous solution, reconstituted recombinant exosomal proteins, CD9, CD63, CD81, ITG αVβ5, or ITG α6β4, were dissolved in 10 mM Tris-Cl buffer (pH 8.0) containing 70 μg/mL siRNA and 13 mM Ca^2+^. The lipid and aqueous solutions were fed into a microfluidic device at a total flow rate of 500 μL/min and flow rate ratio (aqueous phase/lipid phase) of 2. The detailed microchannel structure, fabrication procedure, and experimental setup are described in previously published work.^40^ Exosome-mimicking nanoparticles were collected from an outlet of the microfluidic device and the suspensions were dialyzed against PBS overnight at 4 °C using dialysis membrane tubing (14 kDa MOCW, Repligen Corporation, Waltham, MA). Exosome-mimicking nanoparticles were purified using miniPURE-EV (HansaBioMed Life Sciences Ltd., Tallinn, Estonia), a size extrusion chromatography column, to separate free proteins in the suspension. Each fraction was measured by nanoparticle tracking analysis (NanoSight N300, Malvern Panalytical, Worcestershire, U.K.). The fraction containing nanoparticles was concentrated using Amicon Ultra-4 (100 kDa MOCW, Merck, Germany).

### Preparation of Ionizable Lipid-Composed Exosome-Mimicking Nanoparticles

Ionizable lipid-composed exosome-mimicking nanoparticles were prepared in the same manner as previously described, with minor modifications. SM-102, DSPC, sphingomyelin, DOPE, cholesterol, and DMG-PEG 2k were dissolved in ethanol, and DOPS was dissolved in methanol to prepare the stock solutions. The molar ratio of the lipid solution was SM-102/DSPC/sphingomyelin/DOPE/cholesterol/DOPS/DMG-PEG 2k (20/11/20/5/30/7/1.5), with a total lipid concentration of 8 mM. siRNA and exosomal proteins were dissolved in 25 mM acetate buffer (pH 4.0). The ratio of SM-102 nitrogen to siRNA phosphate was adjusted to 6. For mRNA-loaded exosome-mimicking nanoparticles, luciferase-coded mRNA and ITGs were dissolved in 10 mM citrate buffer at pH 4.0. The ratio of SM-102 nitrogen to mRNA phosphate was adjusted to 9. The other preparation processes were the same as previously described, except for the dialysis conditions. Exosome-mimicking nanoparticles suspensions were dialyzed against 20 mM MES buffer for 2 h, followed by PBS overnight at 4 °C using dialysis membrane tubing.

### Characterization of Exosome-Mimicking Nanoparticles

The size and ζ-potential of exosome-mimicking nanoparticles were measured by a Zetasizer Nano ZS ZEN 3600 (Malvern Instruments, Worcestershire, U.K.). The encapsulation efficiencies of siRNAs and mRNAs were measured by a Ribogreen assay. The presentation of CD9/63/81 was measured by ELISA using commercially available ELISA kits (Hakarel, Ibaraki, Japan) according to the manufacturer’s instructions. ITGs on the exosome-mimicking nanoparticles were determined by flow cytometry (FCM) using SureBeads Protein G Magnetic beads (Bio-Rad, Hercules, CA), as shown in Figure 3. Antihuman ITG αV antibody or antihuman ITG α6 antibody was immobilized onto the magnetic beads according to the manufacturer’s instructions. The magnetic beads were mixed with 0.5 mol % DiD-labeled ITG-decorated exosome-mimicking nanoparticles, followed by three washes with PBS-T to remove unbound antibodies and ITG-negative particles. After washing, 500 μL of FACS buffer was added to the microtubes and the samples were further diluted 20-fold with FACS buffer. The fluorescence of the diluted samples was measured using FCM (CytoFLEX Systems, Beckman Coulter, CA) with excitation and emission wavelengths of 638 and 660 nm, respectively.

### Luciferase Knockdown Assay

HeLa (Hela-dluc) cells (6 × 10^3^ cells per well) expressing firefly and renilla luciferases were cultured in a 96-well microplate for 24 h at 37 °C in 5% CO_2_ prior to transfection. The growth medium consisted of DMEM supplemented with 10% FBS, 100 U/mL penicillin, 100 μg/mL streptomycin, and 400 μg/mL G418 [DMEM (+)]. siRNA-loaded exosome-mimicking nanoparticles were diluted with serum-free DMEM to final concentrations of 1, 20, and 60 nM. HeLa-dluc cells were then treated with 100 μL of the exosome-mimicking nanoparticles for 4 h at 37 °C in 5% CO_2_. After 4 h incubation, the medium was replaced with DMEM (+) and the cells were incubated for an additional 20 h. The luciferase gene knockdown assay was then carried out using the Dual-Glo Luciferase Assay System according to the manufacturer’s instructions.

### Luciferase Expression Assay

HeLa cells were seeded at a density of 4.0 × 10^4^ cells/well in 24-well plates (Thermo Fisher Scientific) and incubated for 24 h at 37 °C in 5% CO_2_. The medium was then removed, and 1 mL of mRNA-loaded exosome-mimicking nanoparticles diluted in DMEM (+) to a final mRNA concentration of 10, 50, or 100 ng/mL were added to each well. The cells were incubated for another 24 h at 37 °C in 5% CO_2_. The wells were washed with PBS, and 100 μL of Glo lysis buffer was added to each well. The lysates were then centrifuged at 15,000 rpm for 2 min at 4 °C. The supernatant was used for the ONE-Glo Luciferase Assay System (Promega) and the BCA Protein Assay (Thermo Fisher Scientific). The luminescence obtained from the ONE-Glo Luciferase Assay System was divided by the total protein amount (mg) in the lysate measured from the BCA Protein Assay to obtain the relative luminescence (RLU/mg protein).

### Cell Viability Assay

HeLa-dluc cells or HeLa cells were cultured in DMEM (+) at 37 °C in 5% CO_2_. Cells were seeded at a density of 6 × 10^3^ cells/well in 100 μL of cell suspension in 96-well clear plates (Thermo Fisher Scientific) and incubated for 24 h at 37 °C in 5% CO_2_. After transfection with siRNA-loaded exosome-mimicking nanoparticles or mRNA-loaded exosome-mimicking nanoparticles, the medium was replaced with 100 μL of DMEM (+). Then, 10 μL of Cell Counting Kit-8 (Dojindo Molecular Technologies, Kumamoto, Japan) was added to each well. Cell viability was measured according to the manufacturer’s protocol.

### Endocytosis Inhibition Assay

To examine the impact of endocytosis inhibition on the cellular uptake and mRNA delivery performance of exosome-mimicking nanoparticles, HeLa cells were pretreated with endocytic inhibitors, including: 20 μM chlorpromazine (CPZ) for clathrin-mediated endocytosis, 50 μM genistein (Gen) for caveolae-mediated endocytosis, 25 mM 5-(*N*-ethyl-*N*-isopropyl) amiloride (EIPA) for macropinocytosis, and 80 μM dynasore (Dyn) for dynamin-dependent endocytosis. One hour prior to transfection, HeLa cells were incubated with DMEM (+) containing the respective inhibitors. After pretreatment, the medium was replaced with DMEM (+) containing 0.5 mol % DiD-labeled (for cellular uptake) or nonlabeled exosome-mimicking nanoparticles (for luciferase expression and cell viability assays) at an mRNA concentration of 100 ng/mL and the corresponding inhibitor. HeLa cells were then incubated for 2 h. The cellular uptake of exosome-mimicking nanoparticles was subsequently quantified by FCM. For the evaluation of luciferase expression and cell viability with endocytosis inhibitors, the medium was replaced with DMEM (+), and HeLa cells were further incubated for 22 h. The luciferase expression level and cell viability were measured in the same manner as previously described. Relative cellular uptake and luciferase expression efficiencies and relative cell viability were normalized to the results obtained without endocytosis inhibitor.

### In Vivo Experiments

The experimental protocols were reviewed and approved by the Hokkaido University Animal Care Committee in accordance with the guidelines for the care and use of laboratory animals. Male and female BALB/c mice (4–8 weeks old) were purchased from Japan SLC (Shizuoka, Japan). The mice were injected via the tail vein with 200 μL of mRNA-loaded exosome-mimicking nanoparticles or mRNA-loaded LNPs (0.05 mg mRNA/kg). After 6 h, the mice received an intraperitoneal injection of 3 mg luciferin and were imaged using an in vivo imaging system (IVIS Lumina III, PerkinElmer, Waltham, MA). For the evaluation of biodistribution, DiR-labeled exosome-mimicking nanoparticles were intravenously administered to mice in the same manner. Ex vivo imaging was performed 3 h postadministration.

### Statistical Analysis

Data are presented as the mean ± SD for the indicated number of experiments. Pair-wise comparisons between treatments were made using a two-tailed Student’s *t*-test. For multiple comparisons, we employed ANOVA, followed by a Bonferroni test.

## Supplementary Material



## Notes

Effect of flow rate ratio on the surface decoration of CD63 on exosome-mimicking nanoparticles, siRNA encapsulation efficiency of exosome-mimicking nanoparticles decorated with tetraspanins, Comparison of CD9 presentation levels on exosome-mimicking nanoparticles produced by the microfluidic device and batch methods; small-angle X-ray scattering (SAXS) analysis of exosome-mimicking nanoparticles, and TEM image of the nanoparticles (PDF)

## #

Department of Chemical Science and Engineering, Tokyo Institute of Technology, Meguro-ku, Tokyo 152-8552, Japan
