# Supplementary material for: Microfluidic Production of Exosome-Mimicking Lipid Nanoparticles for Enhanced RNA Delivery: Role of Exosomal Proteins
Source: ACS Appl Mater Interfaces. 2025 Jun 16;17(29):41666–79. doi: 10.1021/acsami.5c06927 (PMC12292319; doi:10.1021/acsami.5c06927)
Supplement: Supplementary file 1 [file am5c06927_si_001.pdf]

Supporting Information

Microfluidic Production of Exosome-Mimicking Lipid Nanoparticles for Enhanced RNA Delivery: Role of Exosomal Proteins

*Masatoshi Maeki,[a,*1] Ayuka Niwa,[b1] Shota Oyama,[b1] Kyoko Aratani,[c#] Rina Ito,[d] Yuichi Suzuki,[d] Yusuke Sato,[d] Akihiko Ishida,[a] Hideyoshi Harashima,[d] and Manabu Tokeshi[a*]*

[a] *Division of Applied Chemistry, Faculty of Engineering, Hokkaido University, Kita 13 Nishi 8, Kita-ku, Sapporo 060-8628, Japan*

[b] *Graduate School of Chemical Sciences and Engineering, Hokkaido University, Kita 13 Nishi 8, Kita-ku, Sapporo 060-8628, Japan*

[c] *Faculty of Engineering, Hokkaido University, Kita 13 Nishi 8, Kita-ku, Sapporo 060-8628, Japan*

[d] *Faculty of Pharmaceutical Sciences, Hokkaido University, Kita 12 Nishi 8, Kita-ku, Sapporo 060-0812, Japan*

[1] *M.M., A.N., and S.O. contributed equally to this work.*

[#] *Present address: Department of Chemical Science and Engineering, Tokyo Institute of Technology, Meguro-ku, Tokyo 152-8552, Japan*

[*]*Corresponding authors:*

Masatoshi Maeki E-mail address: m.maeki@eng.hokudai.ac.jp

Manabu Tokeshi E-mail address: tokeshi@eng.hokudai.ac.jp

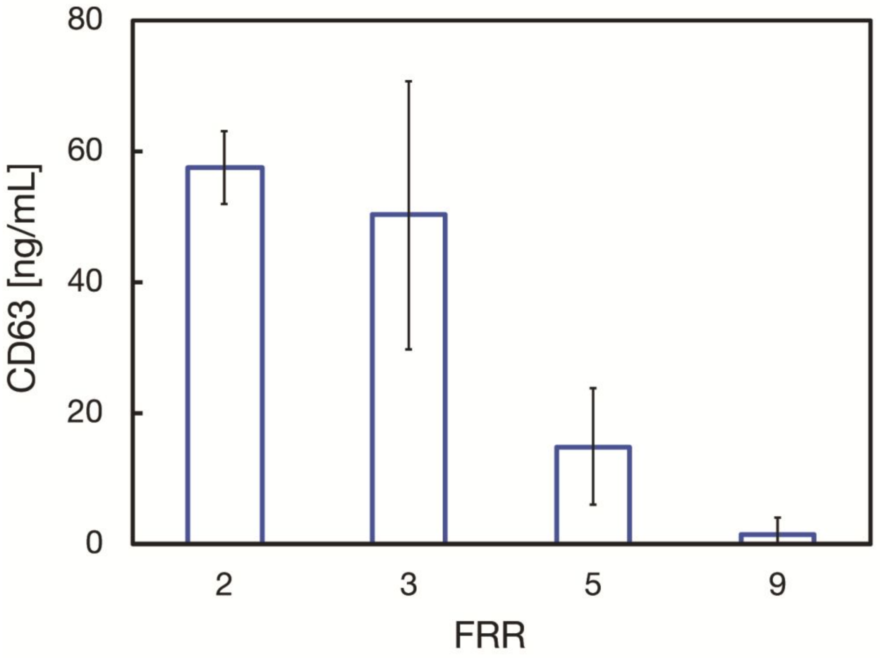

Figure S1. Effect of flow rate ratio on the surface decoration of CD63 on exosome-mimicking nanoparticles. The results of three replicate measurements are shown together with their average and standard deviation (mean ± SD, n = 3).

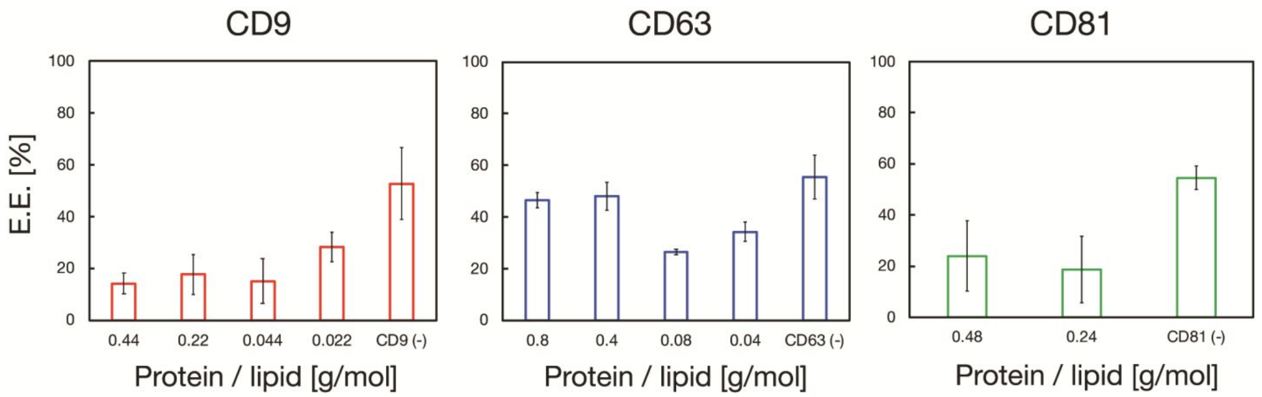

Figure S2. siRNA encapsulation efficiency of exosome-mimicking nanoparticles decorated with CD9, CD63, or CD81. The results of three replicate measurements are shown together with their average and standard deviation (mean ± SD, $n = 3$).

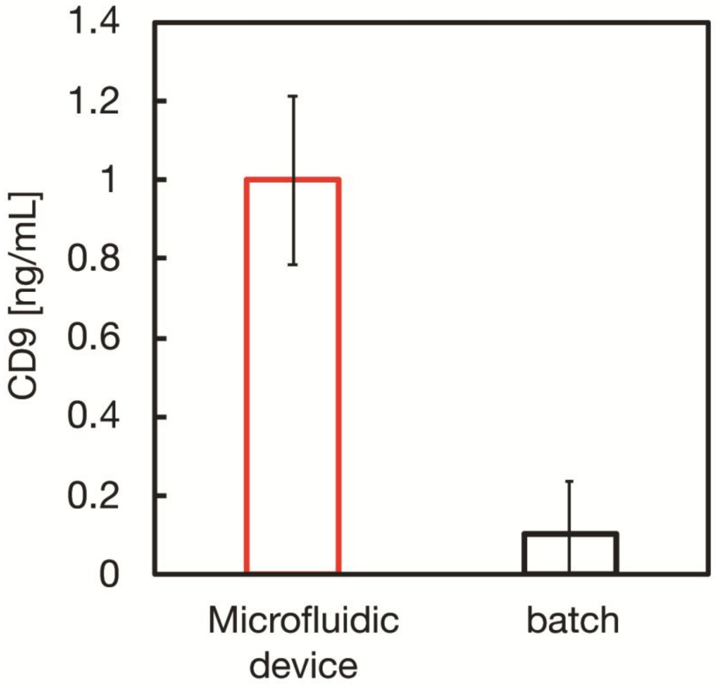

Figure S3. Comparison of CD9 presentation levels on exosome-mimicking nanoparticles produced by the microfluidic device and batch methods. The protein weight to lipid molar ratio was 0.22 g/mol. The results of three replicate measurements are shown together with their average and standard deviation (mean ± SD, $n = 3$).

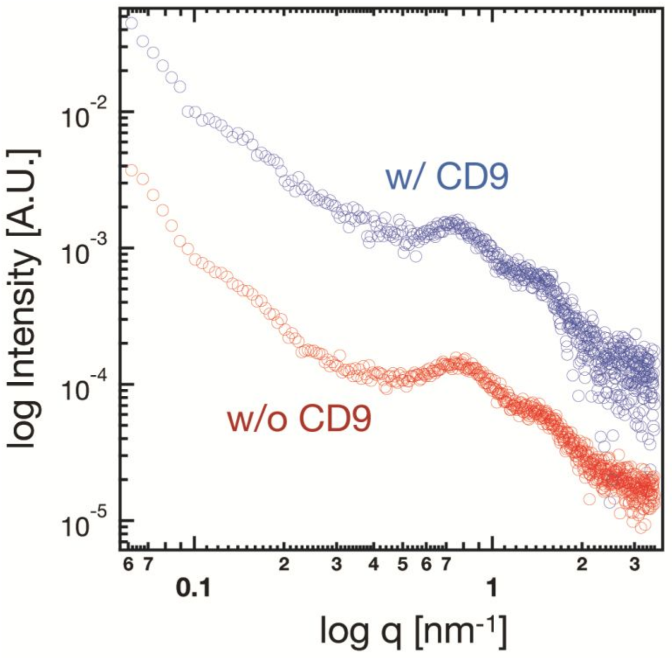

Figure S4. Small-angle X-ray scattering (SAXS) analysis of CD9-decorated exosome-mimicking nanoparticles and CD9 (-) nanoparticles (w/o CD9). SAXS was conducted at beamline BL15A2 at the Photon Factory (High Energy Accelerator Research Organization, Tsukuba, Japan). Each sample was measured using a flow cell, and the SAXS data were measured at a wavelength of 1.213 Å, with the SAXS detector (PILATUS3 X 2M, DECTRIS, Baden, Switzerland) distance set to 1.5 m. SAXS images of each measurement point were collected with exposure times of 1 s, and 1800 images were integrated to generate the SAXS profile. The lamellar d-spacing was calculated to be 8.3 nm from the SAXS peak.

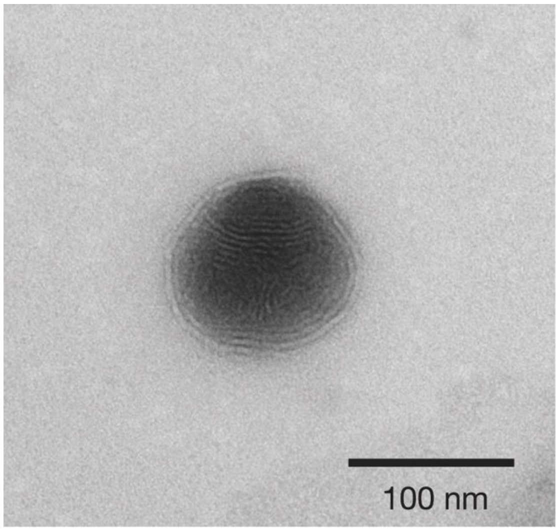

Figure S5. TEM image of the exosome-mimicking particle without exosomal proteins. Exosome-mimicking particles were characterized using TEM (H-7600, Hitachi, Tokyo, Japan) at an acceleration voltage of 100 kV. The nanoparticle suspension was dropped onto a carbon-coated copper grid (400 mesh) and stained with a 2% phosphotungstic acid solution. The TEM images were collected with a charge-coupled device camera (XR16, AMT Imaging, Woburn, MA, USA) at an exposure time of 3.2 s. The nanoparticle size measured by TEM was 100 nm, consistent with DLS measurements. TEM imaging also revealed formation of lamellar structures in the nanoparticles. Scale bar represents 100 nm.
